# Structural and Gas-Sensitive Characteristics of In_2_O_3_: Effect of Hydrothermal/Solvothermal Synthesis Conditions

**DOI:** 10.3390/mi16111299

**Published:** 2025-11-20

**Authors:** Mariya I. Ikim, Varvara A. Demina, Elena Y. Spiridonova, Olusegun J. Ilegbusi, Leonid I. Trakhtenberg

**Affiliations:** 1 N.N. Semenov Federal Research Center for Chemical Physics RAS, 4 Kosygin Street, 119991 Moscow, Russiademina.varvara@yandex.ru (V.A.D.); litrakh@gmail.com (L.I.T.); 2 Department of Mechanical and Aerospace Engineering, University of Central Florida, Orlando, FL 32816, USA; 3 Chemical Faculty, Lomonosov Moscow State University, 119991 Moscow, Russia

**Keywords:** indium oxide, semiconductors, cubic and rhombohedral structures, pores, gas sensor, defect, structural and morphological characteristics

## Abstract

In_2_O_3_ nanoparticles were obtained by annealing precursors that had been hydrothermally/solvothermally synthesized at 200 °C using In(NO_3_)_3_·4.5H_2_O as the starting material. Three solvents were used for the synthesis, namely water, alcohol and ethylene glycol. Urea or glycine additives were introduced into the reaction mixtures as stabilizing and structure-forming agents. The nanopowders obtained were characterized using X-ray diffraction, scanning and transmission electron microscopy, low-temperature nitrogen adsorption and X-ray photoelectron spectroscopy. The gas-sensing characteristics of the indium oxide-based sensors were investigated for the detection of hydrogen in air. It has been established that the nature of the solvent determines the phase composition and structure of indium oxide, while organic additives reduce the particle size and increase the specific surface area. It should be noted that the addition of glycine to an alcohol solution of indium nitrate during synthesis produces a phase transformation. The results show that the sensor based on In_2_O_3_ synthesized using this mixture has the best hydrogen sensing properties of all the materials considered in this study.

## 1. Introduction

Indium oxide (In_2_O_3_) is a semiconductor material with a wide range of applications in modern technologies. Unique physicochemical characteristics, such as high chemical and thermal stability, low activation energy of conductivity, the ability to effectively sorb molecules of various gases and good oxidation–reduction properties, make it an attractive material for the creation of highly efficient gas sensors for various explosive and toxic gases [1,2,3,4,5].

In_2_O_3_ can exist in two polymorphic modifications: a stable cubic structure of the bixbyite type (c-In_2_O_3_) and a metastable rhombohedral structure of the corundum type (rh-In_2_O_3_). The electron affinity of gas molecules is likely to differ across different crystalline phases, which may be useful for the development of new high-performance gas sensors. It should be noted that most of the studies, both experimental and theoretical, in the field of gas sensors have focused mainly on the stable cubic form of indium oxide [6,7,8,9,10]. However, some studies indicate that rhombohedral indium oxide has higher sensory characteristics in the detection of ethanol, ammonia, hydrogen and acetone than its cubic analogue [11,12,13,14,15].

It is still unclear which of the polymorphic modifications of In_2_O_3_ is better, since the sensor characteristics are influenced by several parameters, such as specific surface area, particle size and shape, porosity and the presence of defects. For example, porous nanoparticles of cubic indium oxide demonstrate a higher sensory response to ethanol, formaldehyde and ammonia than microspheres of rhombohedral structure [16]. Various synthesis methods are used to obtain indium oxide nanostructures, including solid-phase, liquid-phase and gas-phase, the conditions of which determine the phase composition, and structural and morphological characteristics of In_2_O_3_ [1].

The hydrothermal/solvothermal approach is often used in sensor studies, since it can synthesize particles with different morphologies and structures by controlling the temperature, pressure and reaction time, as well as the concentration of substances and the type of solvent [17]. For example, titanium oxide nanowires were synthesized using a hydrothermal method at different temperatures [18]. The optimal operating temperature to achieve maximum sensitivity for the detection of 100 ppm ethanol is approximately 180 °C. The hydrothermal method was also used to synthesize SnO_2_ nanorods decorated with bimetallic alloy (Pd-Au) nanoparticles [19]. A high hydrogen response was obtained due to the strong catalytic and synergistic effects of both Pd and Au, and the response and recovery times were 19 s and 302 s, respectively. In addition, the hydrothermal method allows the synthesis of composites such as ZnO/In_2_O_3_ dual metal–organic framework composite for improved detection of ethanolamine [20]. Hydrothermal treatment of an aqueous solution of indium nitrate with the addition of citric acid and urea at temperatures of 120, 160 and 200 °C was found to contribute to the formation of rough microspheres of pure c-In_2_O_3_ nanoparticles, bumpy microspheres of c- and rh-In_2_O_3_ nanoparticles, and porous aggregates of c- and rh-In_2_O_3_ nanocubes and nanoparticles, respectively. It should be emphasized that for the hydrothermal reaction at 120 °C, the synthesis time was increased from 3 to 10 h to bring the size of the cubic In_2_O_3_ microspheres closer to the size of the In_2_O_3_ nanoparticles synthesized at 160 °C [15].

Increasing the hydrothermal/solvothermal synthesis pressure by reducing the free volume in the autoclave of an alcohol solution of indium nitrate with urea leads to an increase in the concentration of the rhombohedral phase, which affects the performance characteristics of the sensor [21]. Polymorphic and morphological transformations of metal oxide can occur as a result of variations in physical and chemical conditions, such as the introduction of additional precursors into the reaction mixtures or changes in the mixture ratio. For example, by using different dopants, the pH of the reaction mixture can be varied. In strongly acidic or alkaline environments, SnO_2_ has a larger surface area, smaller particle size and uniform morphology [22]. The use of dopants thus largely determines the properties and characteristics of SnO_2_ and has a positive effect on its sensitivity to various gases. The addition of different amounts of iron nitrate produces phase transformations of In_2_O_3_ from pure cubic to rhombohedral, and also promotes the formation of a porous lamellar structure [23]. Polymorphic changes were also observed with the combined addition of lanthanum and iron nitrates [24]. The addition of small amounts of polyvinylpyrrolidone produced a noticeable change in morphology from a flower-like shape to one-dimensional nanorods without any phase transformations of indium oxide [25].

There has been a trend in recent years towards the addition of some metals to indium oxide to improve its sensor properties. For example, decorating indium oxide with bimetallic Ag/Cu nanoparticles resulted in a fourfold increase in hydrogen sensitivity [26]. Porous In_2_O_3_ nanotubes co-sensitized with La dopant and Pd modifier produced not only improved response but also a decrease in operating temperature from 160 to 50 °C compared to indium oxide [27]. Despite continued interest in indium oxide-based sensors, there remains a significant research gap. There is no comprehensive study of the influence of solvent and additives during hydrothermal/solvothermal synthesis on the structural, morphological and sensory properties of indium oxide.

The present study involves the synthesis of indium oxide by the hydrothermal/solvothermal method using three solvents, namely water, alcohol and ethylene glycol. Additional agents are also used, namely urea or glycine. The phase composition, structure, conductivity and gas-sensitive characteristics in the detection of 900 ppm H_2_ of the synthesized samples are investigated using various physicochemical methods. The influence of chemical conditions of hydrothermal/solvothermal synthesis on the properties of indium oxide is also analyzed.

## 2. Materials and Methods

Indium nitrate hydrate (In(NO_3_)_3_·4.5H_2_O, 99.9%), urea (99.5%), glycine (Gly, 98.5%), and ethylene glycol (EG, 99%) were purchased from Eco-tec and utilized in this study. Deionized water (H_2_O) and absolute ethanol (C_2_H_5_OH) were also used.

The hydrothermal/solvothermal method was used to synthesize indium oxide nanopowders, utilizing indium nitrate In(NO_3_)_3_·4.5H_2_O as a precursor. The process involved the dissolution of 0.5159 g (1.36 mmol) of indium nitrate in 80 mL of solvent, which is equal to 4.43 mol of distilled water, 1.39 mol of ethanol, and 1.43 mol of ethylene glycol and the addition of 0.9972 g (16 mmol) urea or 1.245 g (16 mmol) glycine. The reaction mixture was sonicated at 30 °C for 15 min, transferred to a 100 mL autoclave, heated at 4 °C/min to 200 °C and held for 2 h to allow the hydrothermal/solvothermal reaction to occur.

After natural cooling of the autoclave to room temperature, the resulting precipitate was separated by centrifugation for 5 min at 4500 rpm, washed several times with water and then dried at 90 °C. Finally, the product obtained was annealed at 500 °C (heating rate of 15 °C/min) for 2 h. The samples were classified according to the following selected solvents and additives: series with water (H_2_O/-, H_2_O/urea, H_2_O/Gly); with ethyl alcohol (C_2_H_5_OH/-, C_2_H_5_OH/urea, C_2_H_5_OH/Gly); and with ethylene glycol (EG/-, EG/urea, EG/Gly).

The phase, structural and morphological characteristics of the synthesized indium oxide nanopowders were determined by X-ray diffraction (XRD), scanning and transmission electron microscopy (SEM and TEM), low-temperature gas adsorption and X-ray photoelectron spectroscopy (XPS). The XRD spectra were recorded using a Rigaku Smartlab SE X-ray (Rigaku, Japan) diffractometer using Cu Kα radiation with a wavelength of 1.5406 Å, with 2θ in the range from 10 to 80° at 0.01° intervals and a scan speed of 5°/min. The particle morphology was studied using SEM on a Thermo Fisher Scientific Prisma (Thermo Fisher Scientific, Waltham, MA, USA) E setup in high vacuum conditions. Images were obtained in secondary electron mode at an accelerating voltage of 5 kV. The particle structure was determined by TEM on a Thermo Fisher Scientific FEI Tecnai Osiris instrument (Thermo Fisher Scientific, Waltham, MA, USA) operating at 200 kV accelerating voltage, equipped with a Fischione HAADF detector (Fischione Instruments, Export, PA, USA) and an EDX microanalysis spectrometer. ImageJ software 1.54h was used to process images and estimate the average particle diameter. XPS spectra characterizing the electronic structure of metal ions on the surface of nanoparticles in the composite were recorded on a Prevac EA15 System spectrometer (Prevac, Rogów, Poland) using Mg(K_α_) radiation (1253.6 eV) as an excitation source. The binding energy of the C1s peak at 284.7 eV was used as a standard to determine the electron binding energies in the samples obtained.

Temperature-programmed H_2_ reduction (H_2_-TPR) and specific surface area measurement were performed on an Altamira AMI-400TPx instrument (Anton Paar, Torrance, CA, USA). The sample was flushed with argon at 300 °C for 1 h to remove surface contaminants and then cooled to room temperature. The sample was then restored to 450 °C at the rate of 10 °C/min using 10% H_2_/Ar at a flow rate of 30 mL/min and subsequently heated. To obtain information on specific surface area, nitrogen and helium were used as the adsorption and carrier gas, respectively, and were introduced into the material in a 30% proportion at a flow rate of 50 mL/min. The specific surface area was calculated using the Brunauer–Emmett–Teller (BET) equation.

**Figure 1:**
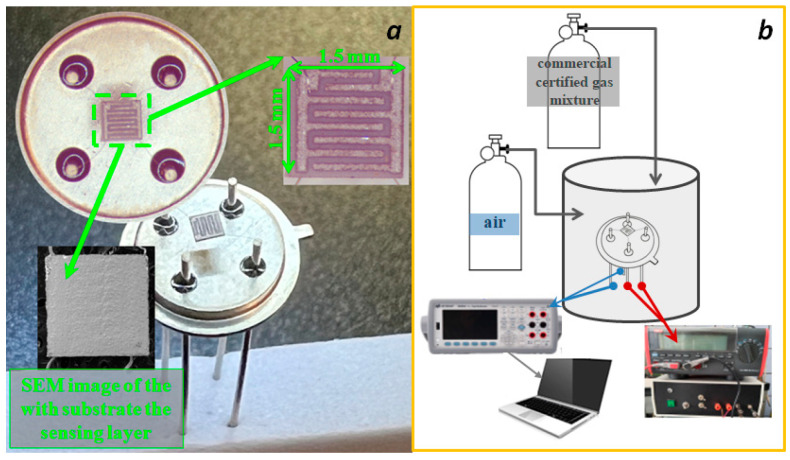
Photograph of the chip and substrate (**a**), and the setup for measuring resistance and sensor response for the detection of various gases (**b**).

To study the conductive and sensory properties, the materials were synthesized on a special chip in the form of a sensitive layer. The chip was an Al_2_O_3_ (polycor) substrate measuring 1.5 mm × 1.5 mm × 0.3 mm with a platinum contact pad on the front side (Figure 1a) and a platinum heater on the back side, which was also used as a thermistor for temperature measurement. The heater resistance at room temperature was 12 ± 1 Ohm. The substrate was attached to the TO5 package using wire leads made of ∅ 20 µm platinum wire in such a way that the substrate was suspended and heat dissipation was achieved through heat exchange with the air (Figure 1a).

The synthesized nanopowders were mixed with a small amount of terpineol and applied as a paste onto the substrate contact pad. Next, the substrate with the applied paste layer was heated for 30 min at a temperature of 100–120 °C. The temperature was then gradually increased to 550 °C until the resistance of the resulting film became constant. A homogeneous film was formed with good adhesion to the substrate as a result of this treatment.

The conductivity and sensor properties were investigated using the developed setup in the temperature range of 300 to 550 °C (Figure 1b). The chip with the applied sensitive layer was placed in a special 1 cm^3^ chamber into which purified air or a commercially available certified gas mixture containing H_2_ or CO in air was supplied. The gas was pumped through the chamber at the rate of 200 mL/min and the temperature was maintained within 1 °C accuracy. The change in sensor resistance was recorded using a Keysight digital multimeter. The signal from the multimeter was transmitted to a computer, where a special program displayed the kinetic curve of resistance variation, reflecting the change in sensor resistance over time when the analyzed gas mixture was introduced. The sensor response (S) was determined from the relation S = R_0_/R_g_, where R_0_ is the initial resistance of the sensor in air, and R_g_ is the minimum value of the sensor resistance achieved after the supply of the analyzed gas.

## 3. Results and Discussion

### 3.1. Structural and Morphological Studies

The influence of solvents and additives on the phase composition and particle size of indium oxide was studied using the XRD method. The nature of the solvent used in hydrothermal/solvothermal synthesis has a significant impact on the composition of both the intermediate and final products. Only peaks from In(OH)_3_ are observed in the XRD spectra of the intermediate products during synthesis in aqueous solutions, corresponding to JCPDS card № 16-0161.

**Figure 2:**
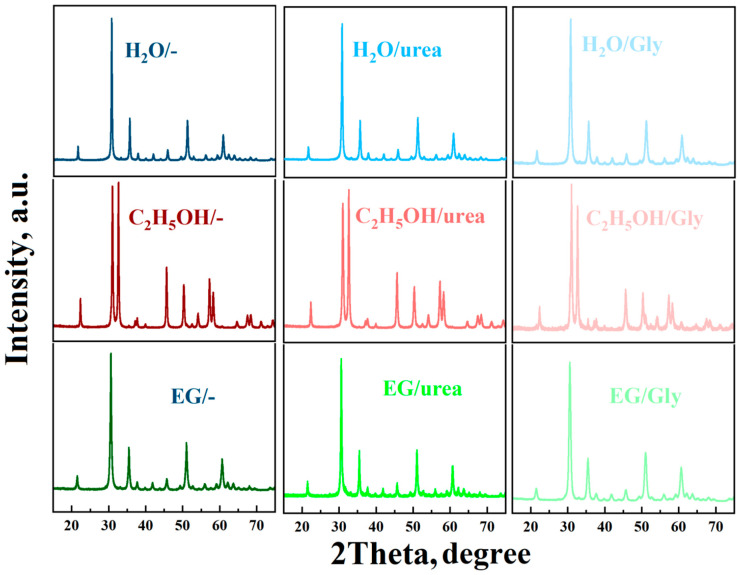
XRD spectra of synthesized indium oxide powders obtained using various solvents and additives.

The solvothermal reaction with ethylene glycol, regardless of additives, produced a gel-like mass that was annealed to form the final product. In addition, regardless of the presence or type of additive during synthesis in water or ethylene glycol, peaks corresponding to JCPDS card № 71-2194 are present in the XRD spectra of the nanopowders obtained (see Figure 2). Specifically, in this case, a cubic phase of indium oxide with a bixbyite structure with a preferred orientation (222) is formed at diffraction angles of 30.54–30.63° (Table 1).

If ethyl alcohol is used as a solvent, intense peaks are observed in the annealed samples in the region of ~31.0° and ~32.6°, corresponding to the (104) and (110) planes of the rhombohedral phase with a corundum structure (JCPDS card № 68-0337) (see Figure 2 and Table 1). The diffraction peaks corresponding to JCPDS card № 17-0549 indicate that the solvothermal reaction product in this case is InOOH. However, the addition of glycine to an alcohol solution of indium nitrate leads to the formation of a small amount of the cubic phase in the sample along with the main rhombohedral phase of indium oxide (Table 1). In addition, a mixture of InOOH and In(OH)_3_ is observed in the XRD spectrum of the solvothermal reaction product. No impurity peaks are observed in the XRD patterns of all the In_2_O_3_ samples obtained, indicating their high purity.

**Figure 3:**
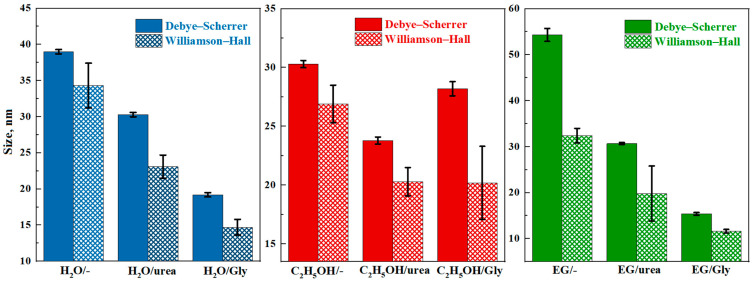
Sizes of indium oxide nanoparticles calculated by the Debye–Scherrer and Williamson–Hall methods.

The sizes of indium oxide nanoparticles in the synthesized powders were calculated from the XRD data using the Debye–Scherrer and Williamson–Hall methods (Figure 3). The Williamson–Hall method, unlike the Debye–Scherrer method, considers the influence of crystal lattice deformation [28]. The data from the main diffraction peak were used for estimating particle size with the Debye–Scherrer method: (222) in the case of a cubic lattice, and (104) in a rhombic lattice. The use of urea or glycine produces a decrease in the size of indium oxide nanoparticles regardless of the solvent type (Figure 3). The particle size of c-In_2_O_3_ synthesized in ethylene glycol is smaller than that of c-In_2_O_3_ synthesized in water with similar additives.

The rh-In_2_O_3_ particles synthesized in alcohol have a smaller size than the cubic phase particles. Figure 3 also presents the crystallite size of the rhombohedral phase for indium oxide synthesized in alcohol with the addition of glycine. The particle size of the cubic phase in this sample is 33.7 nm according to the Debye–Scherrer method. The differences in particle sizes calculated by the two methods indicate the presence of internal stresses in the structure of the synthesized particles.

The phase composition and structure, morphology, and particle size of nanopowders depend on the type of solvents and additives used in hydrothermal synthesis. During the hydrothermal reaction of indium nitrate in the presence of water, In^3+^ is completely hydrolyzed to indium hydroxide, In(OH)_3_

(1) In^3+^ + 3H_2_O ⟹ In(OH)_3_ + 3H^+^

(2) 2In(OH)_3_ ⟹ c-In_2_O_3_ + 3H_2_O

regardless of the nature of the dopant (reaction 1), which decomposes upon calcination, forming indium oxide with a cubic structure (reaction 2). This is confirmed by XRD data.

**Figure 4:**
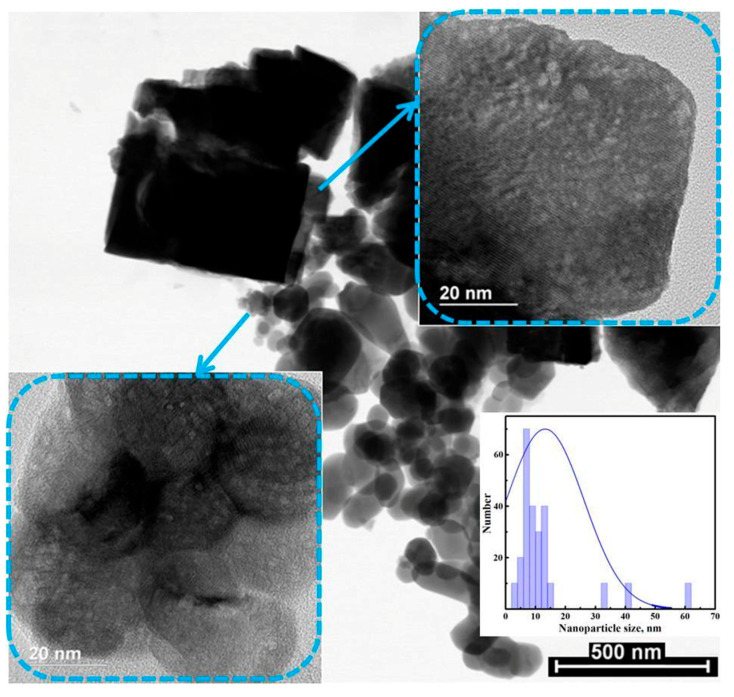
TEM images of sample H_2_O/-.

Hydrothermal synthesis using water as a solvent results in the formation of a mixture of crystallites of various morphologies, both cubic and nearly spherical, with a wide size distribution (Figure 4). The use of additives leads to a slight decrease in particle size, while the specific surface areas increase and are equal to 12, 14 and 19 m^2^/g, respectively, in the cases without the use of additives, and with the addition of urea and glycine.

Urea in the presence of water slowly decomposes with increasing temperature of the hydrothermal reaction

**Figure 5:**
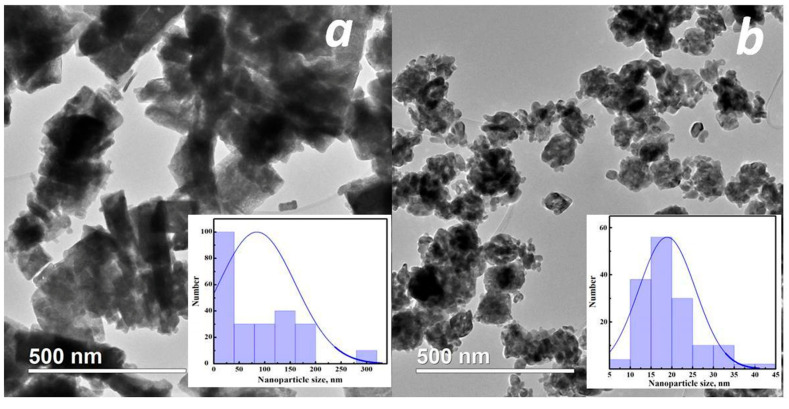
TEM images of samples H_2_O/urea (**a**) and H_2_O/Gly (**b**).

(3) (NH_2_)_2_CO + 3H_2_O ⟹ CO_2_ + 2NH^4+^ + OH^−^

forming ammonium and hydroxide ions, thereby increasing the pH of the solution and reducing the size of indium oxide particles [29]. Similar changes in the size of SnO_2_ particles with changes in the pH of the solution during hydrothermal synthesis have been observed in a previous study [22]. The size of CeO_2_ particles synthesized under hydrothermal conditions also decreased with an increase in the concentration of urea in the solution [30]. In our case, the introduction of urea results in a decrease in the number of individual spherical particles and the formation of aggregates with a dense structure (Figure 5a). The average size of these aggregates is ~170 nm. Most of the aggregates are square, but rectangular ones with a characteristic ratio of 3:1 are also present.

Glycine, one of the simple amino acids, can act as a complexing agent for a number of metal ions because it has a carboxyl group at one end of its molecule and an amino group at the other [31]. Glycine, like urea, does not affect the formation of indium hydroxide as an intermediate reaction product, but it leads to an increase in the viscosity of the solution and, as a consequence, to the formation of non-dense aggregates with dimensions of about 100 nm (Figure 5b).

In the monohydric alcohol ethanol, after synthesis in an autoclave, a precipitate of indium oxyhydroxide InOOH is formed, which at 500 °C decomposes to rhombohedral indium oxide [32,33]:

(4) In^3+^ + C_2_H_5_OH ⟹ In(C_2_H_5_O)_3_ + 3H^+^

(5) In(C_2_H_5_O)_3_ + 3H_2_O ⟹ In(OH)_3_ +3C_2_H_5_OH

(6) In(OH)_3_ ⟹ InOOH + H_2_O

(7) 2InOOH ⟹ rh-In_2_O_3_ + H_2_O

InOOH is formed during the dehydration of In(OH)_3_ due to a lack of water.

When urea is added to alcohol, as with water, it reacts with the solvent, changing the pH of the solution

(8) C_2_H_5_OH + (NH_2_)_2_CO ⟹ C_2_H_5_ONH_2_CO + NH_3_

**Figure 6:**
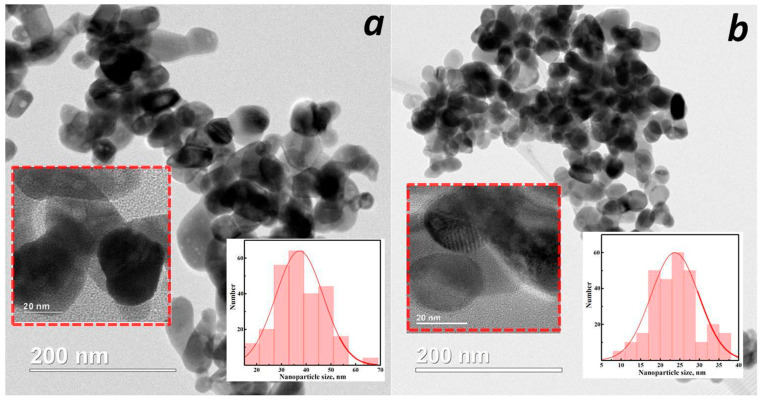
TEM images of C_2_H_5_OH/- (**a**) and C_2_H_5_OH/urea (**b**) samples.

Indium oxide nanoparticles synthesized in alcohol without additives have an irregular shape resembling an ellipse with a length of ~20–30 nm (Figure 6a). Their aggregates are less dense than those synthesized in water, with a branched structure. The specific surface area is 19 m^2^/g. With the addition of urea, the particle shape changes to a spherical shape of ~20 nm (Figure 6b), and the specific surface area increases to 25 m^2^/g.

Ethyl alcohol and glycine undergo an esterification reaction to form the ethyl ester of glycine and water

(9) H_2_N-CH_2_-COOH + C_2_H_5_OH ⟹ H_2_N-CH_2_-COO-C_2_H_5_ + H_2_O

which leads to the formation of not only indium oxyhydroxide but also indium hydroxide (reactions 1–2). The formation of two phases was observed upon the addition of water to DMF [34]. Further annealing of the resulting precipitate of a mixture of indium hydroxide and oxyhydroxide results in a mixture of rhombohedral and cubic phases.

**Figure 7:**
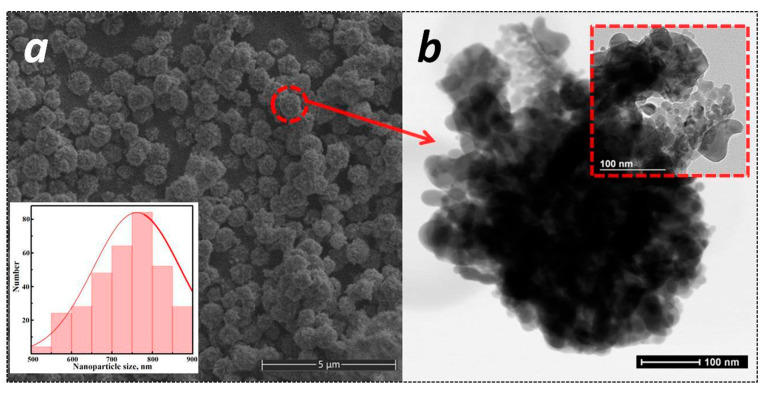
SEM- (**a**) and TEM (**b**) images of C_2_H_5_OH/Gly sample.

Of particular interest is the morphology of indium oxide particles obtained in alcohol with the addition of glycine. The synthesis resulted in the formation of highly monodisperse spherical aggregates, approximately 800 nm in size, consisting of particles ~20 nm in size (Figure 7). The specific surface area is 16 m^2^/g.

**Figure 8:**
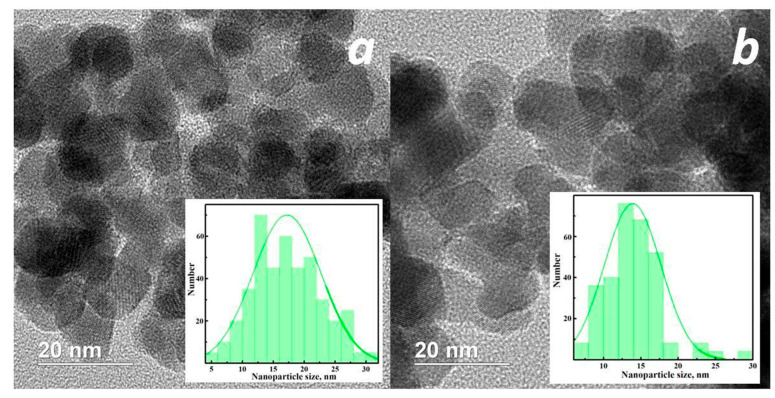
TEM images of samples EG/- (**a**) and EG/Gly (**b**).

Indium oxide powders synthesized in ethylene glycol exhibit the highest specific surface area. The specific surface areas are 26, 33, and 36 m^2^/g for the powders without additives, and with the addition of urea and glycine, respectively. In all samples synthesized using EG, the particles are spherical with a narrow size distribution. The particles form aggregates, but not dense ones, with a branched surface (Figure 8). As noted earlier, no precipitate was formed after the solvothermal reaction, and the intermediate product had a gel-like structure.

Thus, this morphology of indium oxide particles, as well as the increase in the specific surface area compared to other samples, can be explained by the formation of pores during thermal destruction of the gel. It is likely that an In-EG complex is formed during the initial stage of the solvothermal reaction, which polymerizes with increasing temperature. EG serves not only as a solvent but also, most likely, as a surfactant, influencing the formation of the spherical morphology of the nanoparticles. The addition of urea and glycine in this case facilitates faster and more uniform nucleation of nanoparticles from the complex precursor [35].

The spatial distribution of indium and oxygen in indium oxide powders was studied using EDX mapping of the corresponding elements. The distribution of these elements was uniform across all samples, and no impurity elements were detected.

**Figure 9:**
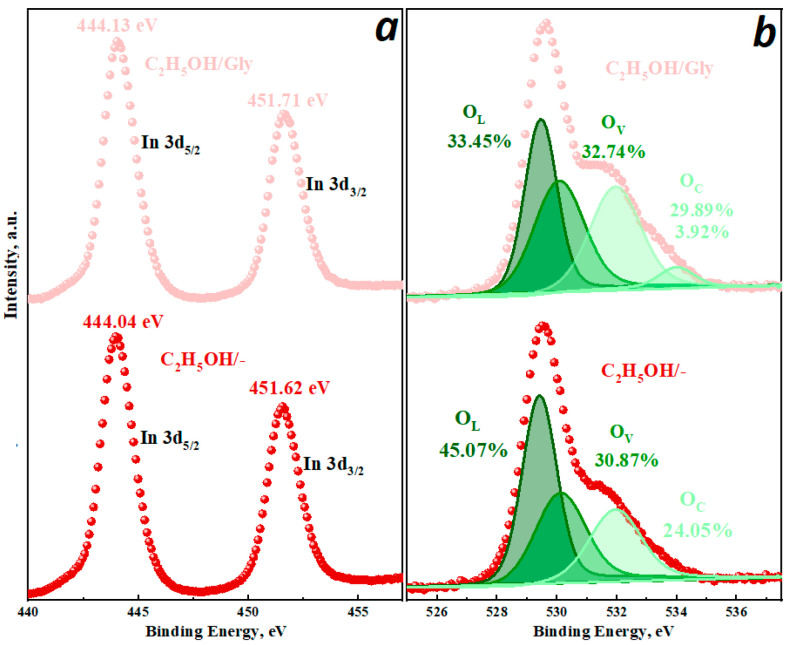
High-resolution XPS spectra of In 3d (**a**) and O 1s (**b**).

To determine the chemical composition, valence states of surface elements and active oxygen centers, samples were analyzed using the XPS method. The survey spectra exhibit characteristic peaks of In, O, C without any other impurity elements, indicating high purity of the synthesized samples. The high-resolution spectra of In 3d show two intense peaks at binding energies of around 444 and 452 eV, corresponding to In 3d5/2 and In 3d3/2, respectively, indicating the indium +3 valence state. The binding energies of In 3d electrons in the C_2_H_5_OH/Gly sample are shifted to a position with higher binding energy compared to C_2_H_5_OH/- (Figure 9a).

The XRD results show that the introduction of glycine into the reaction mixture leads to the formation of two polymorphic modifications of indium oxide. The observed energy shifts in the In 3d peaks also indicate the formation of a heterojunction between the cubic and rhombohedral phases in the C_2_H_5_OH/Gly sample. The XPS O 1s were also analyzed due to the significance of the chemical state of oxygen to the sensor performance (Figure 9b). The asymmetric O 1s peak can be resolved into several peaks: lattice oxygen (OL, ~529.5 eV), oxygen vacancies (OV, ~531.1 eV), and various forms of chemisorbed oxygen (OC, ~532.7 eV). In addition, the concentration of various forms of oxygen in the samples depends on the synthesis conditions, which determine the phase composition, morphology, and structure of indium oxide. Thus, the introduction of additives used in this study in all series leads to an increase in the concentration of oxygen vacancies. Higher O_V_ content generally means higher performance in sensor applications. Note that a slight decrease in the number of O_C_ is observed in the H_2_O/urea sample.

Thus, the proposed chemical reactions explain the formation of polymorphic modifications of indium oxide depending on the type of solvent. In addition, an interpretation is provided of the role of urea (pH change) and glycine (complexation, viscosity change, esterification) in controlling particle size and morphology. The relationship between the structure of indium oxide and its surface chemical composition is also explained.

### 3.2. Conductivity and Sensor Response to Hydrogen

**Figure 10:**
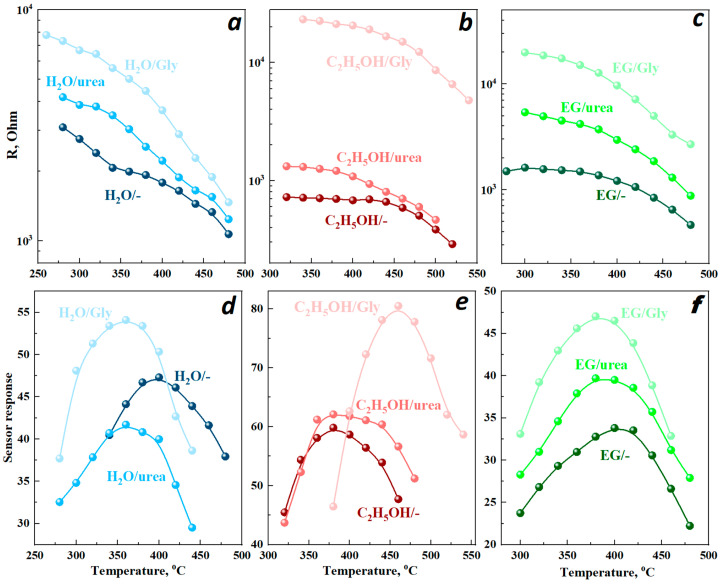
Temperature dependence of resistance of samples synthesized in H_2_O (**a**), C_2_H_5_OH (**b**), EG (**c**) and sensor response to 900 ppm H_2_ in air (30% RH): (**d**,**e**,**f**), respectively.

The influence of the type of solvent or additive used in the synthesis of indium oxide nanopowders was assessed, considering the data on the resistance of the sensitive layers based on them. Figure 10a–c show that in the range from 300 to 500 °C, the resistance of all samples decreases with increasing temperature, regardless of the synthesis conditions, which is typical for *n*-type semiconductors. This is due to the increase in charge carrier concentration in the semiconductor with increasing temperature.

**Figure 11:**
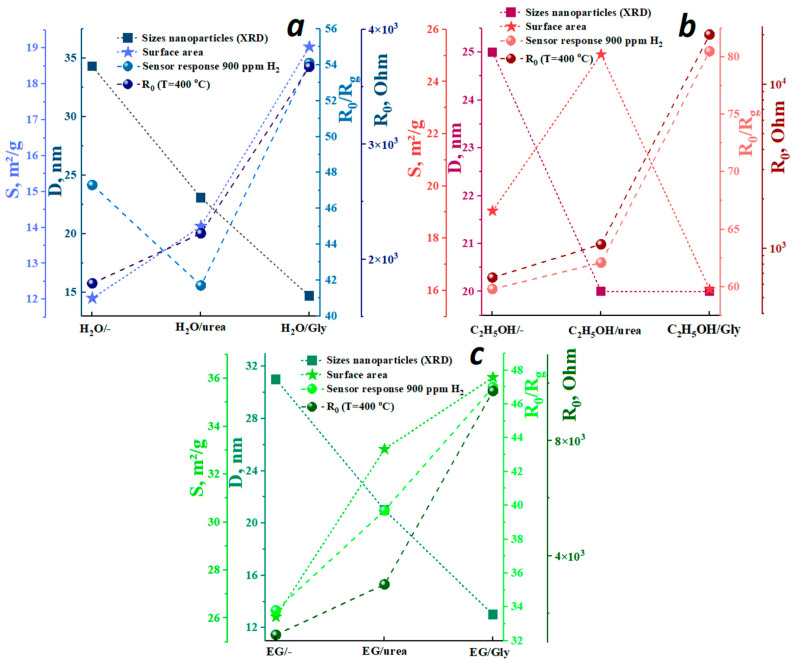
Correlation between surface area, crystallite size, and resistance in air, sensor response to 900 ppm H_2_ of samples synthesized in H_2_O (**a**), C_2_H_5_OH (**b**), EG (**c**).

Using different solvents, the resistance values at the same temperatures follow the sequence H_2_O/- (1.78 kOhm at 400 °C) > EG/- (1.2 kOhm at 400 °C) > C_2_H_5_OH/- (0.28 kOhm at 400 °C). The introduction of additives in all cases leads to an increase in the resistance of the films in air (Figure 10a–c and Figure 11). The synthesis in water and ethylene glycol produces cubic indium oxide with different morphological characteristics. The more developed surface of the EG/- sample (26 m^2^/g) compared to H_2_O/- (12 m^2^/g) is characterized by a larger number of surface-adsorbed forms of oxygen that capture electrons from the conduction band, thereby increasing the resistance.

The additives introduced in samples synthesized using ethylene glycol do not change the surface morphology but merely result in a reduction in particle size. A comparison of the data in Figure 11c indicates that the resistance increases with decreasing particle size. This trend is attributed to the increased contribution of intercrystalline boundaries to the impediment of charge carrier transport. In addition, introducing additives into the aqueous solution not only reduces the size but also slightly alters the shape of the indium oxide nanoparticles, which also contributes to an increase in resistance.

Samples synthesized using ethyl alcohol as a solvent have a rhombohedral structure and exhibit lower resistance than the samples with a cubic structure. The rh-In_2_O_3_ structure, unlike c-In_2_O_3_, contains elongated and weaker In-O bonds, which make it easier to create bulk defects, such as oxygen vacancies [36]. The formation of a vacancy changes the electron distribution around it, which increases the concentration of conduction electrons. The introduction of urea into the reaction mixture does not produce phase changes in the indium oxide, but merely reduces the particle size, which slightly increases the resistance of the sample (Figure 10b).

When glycine is used in the sample, a cubic phase of indium oxide is formed along with the rhombohedral phase, producing a sharp increase in resistance (Figure 10b). This result can be explained primarily by the presence of heterojunctions between c- and rh-In_2_O_3_. An increase in resistance is also observed in systems based on In_2_O_3_ with a number of other contacting metal oxides, with the formation of an *n*-*n* heterojunction [37,38,39].

The sensor properties of sensitive layers based on the synthesized indium oxide nanopowders for detecting 900 ppm hydrogen in air were investigated in the temperature range of 250 to 550 °C (Figure 10d–f). The response to hydrogen is characterized by an increase in the conductivity of the films as a result of the reaction of hydrogen with oxygen acceptor centers [40]. In the temperature range of 200–500 °C, the active centers are oxygen anions (O^−^) [41], and during the sensor reaction, the conduction electrons captured by oxygen are released. The response reaches its maximum value at a temperature determined by the rates of formation and destruction of active reaction centers on the surface of In_2_O_3_ nanoparticles.

Based on the data obtained, sensory properties depend on the structure and morphology of the samples, which in turn are determined by the synthesis conditions (Figure 10d–f). The operating temperature of the samples synthesized using ethylene glycol is in the range of 380–400 °C and is independent of the additives introduced. The maximum sensor response to hydrogen for these samples increases due to the reduction in particle size caused by the additives. This size reduction creates more active sites for hydrogen adsorption. Notably, the addition of urea and glycine to an aqueous indium nitrate solution results in a 60 °C reduction in the operating temperature for hydrogen detection (Figure 10d).

The effects of the additives on sensory response vary (Figure 11). For example, the addition of urea to an aqueous solution causes a decrease in hydrogen sensitivity, while glycine increases it, with a decrease in particle size and an increase in specific surface area (Figure 11a). This trend is most likely due to the textural characteristics of the synthesized samples. Since the use of water and ethylene glycol as solvents results in the formation of a cubic phase of indium oxide, the values of the sensory response to hydrogen in these series are similar. Samples with a rhombohedral structure exhibit the highest sensory response to 900 ppm hydrogen, equal to 80.5. This result is due to the fact that *rh*-In_2_O_3_ nanoparticles contain a higher concentration of active sites for the sensory reaction of hydrogen with oxygen on their surface than c-In_2_O_3_. Moreover, these centers are more labile and therefore more active. This implies that the surface of rh-In_2_O_3_ nanoparticles is more active in sensory reactions than the surface of c-In_2_O_3_ (Figure 10d–f).

The data obtained indicates that the introduction of urea into an alcohol solution leads to a decrease in the particle size and an increase in specific surface area without changing their morphological characteristics (Figure 11b). This, in turn, contributes to a slight increase in the response to hydrogen at the same operating temperature of 380 °C (Figure 10e). Glycine produces the formation of spherical agglomerates consisting of nanoparticles of the c- and rh-In_2_O_3_ phases (Figure 7). An *n-n* heterojunction is formed at the interface as a result of contact between cubic and rhombohedral In_2_O_3_ nanoparticles. In this case, the electron work function and its difference between c-In_2_O_3_ and rh-In_2_O_3_ nanoparticles have a significant impact on the resistance and sensor response of the samples. It is obvious that electrons will begin to flow from rh-In_2_O_3_ to c-In_2_O_3_, since the electron work function for the rhombohedral phase, which is equal to 4.3 eV, is less than for the cubic phase (5 eV) [42]. An electron-depleted layer is formed at the interface in rh-In_2_O_3_ while the electron concentration increases in c-In_2_O_3_. The oxygen adsorption thus increases, accompanied by the formation of a negatively charged O^−^ layer. All this contributes to an increase in the resistance of the samples (Figure 10b and Figure 11b).

The performance of In_2_O_3_-based sensors in detecting reducing gases is closely related to the catalytic processes on the surface of the sensing layer and the electron transfer caused by the sensor reaction. Specifically, when the In_2_O_3_-based sensitive layer is exposed to air, electrons from the conduction band of indium oxide are captured by oxygen particles, forming chemisorbed oxygen ions on the surface. This process helps to reduce the electron concentration in In_2_O_3_, which leads to an increase in the resistance of the sensitive layer. If hydrogen appears in the system, H_2_ molecules are adsorbed on the In_2_O_3_ surface and then undergo a redox reaction with previously chemisorbed oxygen ions

(10) H_2_ + O^−^(ads) ⇄ H_2_O(gas) + e^−^

where the O^−^ layer disappears, the electrons return to the volume of the nanoparticle, and the resistance of the sensitive layer decreases.

Obviously, the influence of *n-n* heterojunction on the sensor efficiency depends on the ratio (*R*_0_)|_H2≠0_/*n_c_*(*R*_0_)|_H2=0_, where *n_c_*(*R*_0_) is the concentration of conducting electrons near the surface of the nanoparticle. Thus, it is necessary to compare the value of this ratio for a sensor with and without a heterojunction. In this study, the presence of a heterojunction increases the sensory effect (Figure 11b), but can also decrease it. Using the methods presented in previous studies [43,44], the heterojunction influence on the sensor response for a specific sensory system can be calculated.

### 3.3. Catalytic Activity of Nanopowders

The temperature-programmed reduction (TPR) technique was used to evaluate the catalytic activity of the synthesized indium oxide nanopowders. The redox reaction between H_2_ and the surface of various In_2_O_3_ structures was investigated. Peaks at temperatures above 300 °C correspond to the bulk reduction of In_2_O_3_ to metallic indium [45]. Peaks at temperatures below 300 °C correspond to the reduction of various forms of chemisorbed oxygen, as well as the formation of surface oxygen vacancies.

**Figure 12:**
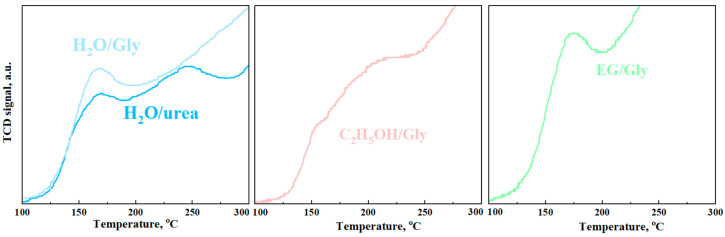
TPR-H_2_ curves In_2_O_3_ nanopowders.

The temperature of complete reduction of cubic indium oxide synthesized using water or ethylene glycol is in the range of 405–416 °C, which is lower than that of rhombohedral indium oxide of about 495–509 °C. Such a difference in the reduction temperatures of polymorphic modifications of indium oxide was also observed in a previous study [46]. Figure 12 shows the characteristic H_2_-TPR profiles for each series of synthesized indium oxide nanopowders in the temperature range of 100–300 °C.

In the low-temperature region, surface reduction peaks for rhombohedral indium oxide are observed at higher temperatures compared to cubic samples. It is worth noting that the H_2_-TPR profile of the H_2_O/- and H_2_O/urea samples exhibits two peaks at about 160 °C and above 200 °C. The peak above 200 °C corresponds to the reduction in rectangular particles observed in the samples (see Figure 4). It has been shown that hydrogen reduction peaks on the In_2_O_3_ surface with three different morphologies occur at different temperatures [47].

The introduction of additives in each of the series of samples leads to a decrease in particle size (Figure 11), which in turn contributes to an increase in the defectiveness of the surface layers. This is also accompanied by a slight decrease in the hydrogen reduction temperature (Table 2).

The amount of H_2_ consumed in samples with a rhombohedral phase is almost double that of the cubic phase (Table 2). It should be noted that for samples obtained in ethylene glycol solutions, hydrogen consumption is lower (0.42–0.48 mmol/g) than for nanopowders obtained from aqueous solutions (0.32–0.35 mmol/g) with the same phase structure. By comparing the data on the maximum sensory response to hydrogen with its consumption during the restoration of the surface of the samples, a good correlation can be found (Table 2). In the low-temperature region, oxidation–reduction reactions occur between H_2_ and chemisorbed oxygen ions on the surface of the samples. The stronger the reactions, the higher the catalytic activity of the surface, and correspondingly, the higher the response to hydrogen.

### 3.4. Sensor Characteristics

**Figure 13:**
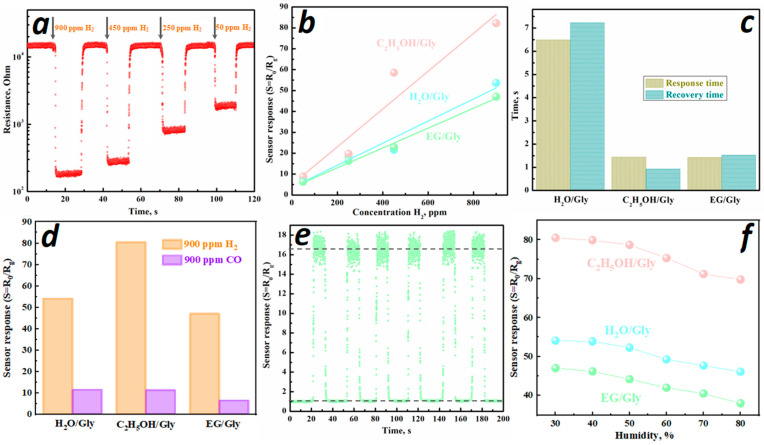
Dynamic response and recovery curve of C_2_H_5_OH/Gly on exposure to 50–900 ppm of H_2_ (**a**). Dependence of the sensor response of synthesized samples on H_2_ concentration (**b**). Response/recovery times for detection of 900 ppm H_2_ (**c**). Sensor response of samples to 900 ppm H_2_ and CO in air (**d**). The EG/Gly sensor response cyclic (250 ppm H_2_, 30% humidity) (**e**). Sensor response of synthesized samples under different humidity levels (**f**).

To study the sensory characteristics, samples synthesized using glycine were selected: H_2_O/Gly, C_2_H_5_OH/Gly, and EG/Gly. These samples demonstrated the best sensor response to 900 ppm H_2_ in each of the series considered. This trend is associated with the smaller particle size, an increase in the specific surface area, and higher catalytic activity of the surface. In the case of C_2_H_5_OH/Gly samples and with a change in phase composition, a rhombohedral phase of indium oxide is formed along with the cubic phase. Figure 13a shows the dynamic resistance curves of C_2_H_5_OH/Gly for the detection of different H_2_ concentrations ranging from 50 to 900 ppm at an operating temperature of 460 °C and 30% humidity. The sensor resistance gradually increases with a reduction in hydrogen concentration in the air and remains stable (Figure 13a).

As the hydrogen concentration increases, the response value for all samples studied proportionally increases (Figure 13b). The samples exhibit a linear response dependence on the H_2_ concentration, with slopes of 0.96 for H_2_O/Gly, 0.93 for C_2_H_5_OH/Gly, and 0.99 for EG/Gly. The detection limit was calculated using the formula:

(11) LOD = 3σ/S

where σ and S are the standard deviation of the sensor resistance and the slope [48], respectively. The theoretical detection limits of H_2_ for the synthesized samples are 323 ppb—H_2_O/Gly, 173 ppb—C_2_H_5_OH/Gly, and 317 ppb—EG/Gly. It should be noted that all the samples investigated are characterized by a relatively low limit and a wide range of hydrogen detection, since the sensors do not reach saturation at hydrogen concentrations up to 900 ppm (Figure 13b).

The sensor response/recovery time was determined using the dynamic resistance curves of the sensor as the time required to achieve a 90% change in sensor resistance upon the introduction and removal of hydrogen. The response and recovery times for hydrogen concentration of 900 ppm are 6.49 and 7.23 s for the H_2_O/Gly sample, 1.44 and 0.92 for the C_2_H_5_OH/Gly sample, and 1.42 and 1.52 for the EG/Gly sample, respectively (Figure 13c).

The selectivity of metal oxide sensors is one of the most important parameters determining their practical value. To evaluate the selectivity of the synthesized systems, CO and H_2_, which exhibit high cross-sensitivity, were chosen as target gases (Figure 13d). All the sensors investigated demonstrated a higher response to hydrogen than to CO, indicating their selectivity for H_2_. The S_H2_/S_CO_ selectivity coefficient was 4.7 for the H_2_O/Gly sample, 7.1 for the C_2_H_5_OH/Gly sample, and 7.2 for the EG/Gly sample. Furthermore, the theoretical detection limit for CO, calculated using the formula in Equation (11), is 1.39 ppm for H_2_O/Gly, 1.26 ppm for C_2_H_5_OH/Gly, and 2.25 ppm for EG/Gly. Thus, the sensitivity of sensors based on all the synthesized systems to hydrogen is higher than to carbon monoxide.

Stability and reproducibility are also important parameters for the assessment of the performance of gas sensors. Figure 13e shows the response curve for the EG/Gly sample at 250 ppm H_2_. After six consecutive cycles, neither the response value nor the response curve characteristics changed, demonstrating excellent reproducibility. To study long-term stability, the resistance and response of the sensors to hydrogen were checked every five days for three months. The resistance of the samples in air and their response did not change by more than 5–7% over that period, indicating the stability of the samples.

It was also found that increasing the humidity from 30 to 80% caused a gradual decrease in the sensory response to 900 ppm H_2_ for all the samples (Figure 13f). This effect is due to the filling of active sites on the surface of the material with water molecules, which reduces the proportion of the reaction between hydrogen and adsorbed oxygen.

Table 3 presents the hydrogen detection sensing properties of different In_2_O_3_ samples synthesized by the hydrothermal/solvothermal method using various additives and solvents. The additives and solvents used in the same synthesis method were found to produce different sensor characteristics of indium oxide (Table 3). Our samples have higher sensor response values to H_2_ compared to data obtained from the literature, but at a higher operating temperature. However, a comparison of a sensor based on cubic indium oxide [49] with H_2_O/Gly, for example, shows that even at low temperatures, our sample will have a higher response to virtually the same hydrogen concentration (see Figure 10a).

It is also worth noting that samples consisting of a mixture of cubic and rhombohedral indium oxide phases exhibit superior performance compared to the pure cubic phase. This is confirmed not only by the present work but also by the results of a previous study [50]. A comparison with existing data shows that our samples also exhibit shorter response/recovery times, low hydrogen detection limit, and reasonable selectivity for CO (see Table 3). Thus, our sensors have promising applications in both industrial safety and environmental monitoring.

## 4. Conclusions

In_2_O_3_ nanopowders were synthesized using a hydrothermal/solvothermal method. It was shown that the choice of solvent and additives significantly affects the phase composition, structure, particle size, and sensor properties of indium oxide. The solvents used in the study have a significant impact on the phase composition of the intermediate and final products. A hydrothermal reaction in an aqueous solution results in the formation of indium hydroxide, which is converted to cubic indium oxide. The use of ethylene glycol as a solvent produces a gel-like mass and the formation of c-In_2_O_3_ during annealing. The alcohol solution leads to the formation of indium oxyhydroxide, which transforms into rhombohedral indium oxide.

Urea and glycine help reduce the particle size of indium oxide in all solutions used. However, these additives also alter the textural properties of indium oxide in aqueous solutions. Along with changes in particle morphology and size, the addition of glycine to the alcohol solution promotes the formation of a mixture of c-In_2_O_3_ and rh-In_2_O_3_ phases. The rhombohedral phase nanoparticles are characterized by the smallest size of all the cases considered. It has been established that the resistance value of In_2_O_3_ under identical temperature conditions decreases in the following order: water, ethylene glycol, and alcohol.

Regardless of the type of solution, the introduction of organic additives results in an increase in the resistance of the sensitive layer. The introduction of glycine into an alcohol solution forms a mixed polycrystalline phase, and the system’s resistance increases sharply due to the formation of heterojunctions. The operating temperature of ethylene glycol-based samples was found to be 380–400 °C and is independent of additives. The increase in sensor response is associated with a decrease in particle size and the creation of new active sites for hydrogen adsorption.

The introduction of additives to the aqueous solution reduces the operating temperature by approximately 60 °C, with urea reducing sensitivity and glycine increasing it. The highest sensitivity is characteristic of the rhombohedral phase of indium oxide due to the increased number of active sites on the surface. The best sensor response to hydrogen among all the synthesized systems was demonstrated by the sample consisting of two polymorphic modifications of indium oxide. The sample also has an interesting structure, consisting of nanospheres composed of a mixture of particles. A correlation has been established between the catalytic activity of the surface and the sensor response, providing a valuable experimental basis for studying gas-sensitive metal oxide materials.

The results presented demonstrate that the choice of solvents and additives in hydrothermal/solvatothermal synthesis enables rapid control of the structure, morphology, as well as chemical and electrical properties of indium oxide. This approach can achieve gas sensor performance comparable to that achieved with the addition of precious metals. Furthermore, the synthesis time will be significantly reduced, thereby lowering the cost of the sensors.

In addition to structural and morphological studies, the study of catalytic activity using the TPR method, which is rarely used to describe the sensor’s operating mechanism, is of particular interest. It has been shown that this method allows for simple and rapid response interpretation compared to other gas sensor characterization methods and can be applied to other metal oxide-based sensors.

## Figures and Tables

**Table 1:** XRD data for indium oxide powders.

Sample	Crystalline Phase, wt%	2θ, °	Lattice Parameters, nm	d-Spacing, nm
H_2_O/-	100% c-In_2_O_3_	30.63 (222)	a = b = c = 1.0106	0.2916 (222)
H_2_O/urea	100% c-In_2_O_3_	30.61 (222)	a = b = c = 1.0111	0.2918 (222)
H_2_O/Gly	100% c-In_2_O_3_	30.59 (222)	a = b = c = 1.0116	0.2920 (222)
C_2_H_5_OH/-	100% rh-In_2_O_3_	30.98 (104) 32.61 (110)	a = b = 0.5483; c = 1.4502	0.2885 (104) 0.2743 (110)
C_2_H_5_OH/urea	100% rh-In_2_O_3_	31.00 (104) 32.61 (110)	a = b = 0.5486; c = 1.4507	0.2884 (104) 0.2744 (110)
C_2_H_5_OH/Gly	89.2% rh-In_2_O_3_	31.00 (104) 32.64 (110)	a = b = 0.5484; c = 1.4508	0.2882 (104) 0.2742 (110)
	10.8% c-In_2_O_3_	30.63 (222)	a = b = c = 1.0117	0.2917 (222)
EG/-	100% c-In_2_O_3_	30.54 (222)	a = b = c = 1.0118	0.2924 (222)
EG/urea	100% c-In_2_O_3_	30.58 (222)	a = b = c = 1.0122	0.2925 (222)
EG/Gly	100% c-In_2_O_3_	30.54 (222)	a = b = c = 1.0119	0.2921 (222)

**Table 2:** The results of the TPR-H_2_ experiments and the maximum sensor response.

Sample	Low Temperature Peaks, °C	Consumption of H_2_, mmol/g	Maximum Sensor Response
H_2_O/-	158 265	0.43	47.3
H_2_O/urea	156 234	0.42	41.8
H_2_O/Gly	164	0.48	53.6
C_2_H_5_OH/-	207	0.79	59.8
C_2_H_5_OH/urea	206	0.8	62.1
C_2_H_5_OH/Gly	210	1.34	80.5
EG/-	177	0.32	33.8
EG/urea	173	0.34	39.6
EG/Gly	170	0.35	47

**Table 3:** Sensor characteristics to various concentrations of hydrogen of polymorphic modifications of In_2_O_3_.

In_2_O_3_ Phase	Materials for Synthesis	S at [H_2_]	T, °C	t_res_/t_rec_, s	LOD	S_H2_/S_CO_	Ref.
c-In_2_O_3_	0.5 g of In(NO_3_)_3_ and 9 g of C_6_H_5_Na_3_O_7_, 1.5 g of CO(NH_2_)_2_ were dissolved in 60 mL water	2.3 * at 100 ppm, 3 at 200 ppm	300	24/25	-	2	[26]
c-In_2_O_3_	0.6 g of In(NO_3_)_3_ and 0.3 g of H_2_BDC were dissolved in 60 mL DMF	1.72 * at 50 ppm	160	74/259	-	1.6	[27]
c-In_2_O_3_	409.12 mg In(NO_3_)_3_ and 996.98 mg urea were dissolved in 80 mL ethanol	1.62 * at 1000 ppm	160	-	5 ppm	1.03	[49]
c+rh-In_2_O_3_	0.5 mmol In(NO_3_)_3_ and 0.5 mmol CO(NH_2_)_2_, 0.1 mmol SDS were dissolved in 20 mL ethanol and water	111.6 ** at 200 ppm	175	5/8	-	13	[50]
c-In_2_O_3_	0.5 mmol In(NO_3_)_3_ and 0.5 mmol CO(NH_2_)_2_, 0.1 mmol SDS were dissolved in 20 mL absolute and water	10.2 ** at 200 ppm	175	9/10	-	-	[50]
c-In_2_O_3_	1.36 mmol In(NO_3_)_3_ and 16 mmol Gly were dissolved in 80 mL water	53.6 * at 900 ppm	360	6.5/7.2	323 ppb	4.7	This work
c+rh-In_2_O_3_	1.36 mmol In(NO_3_)_3_ and 16 mmol Gly were dissolved in 80 mL ethanol	80.5 * at 900 ppm	480	1.4/1	173 ppb	7.1	This work

* S = R_0_/R_g_; ** S = (R_0_ − R_g_)/R_g_ × 100.

## Data Availability

The original contributions presented in this study are included in the article. Further inquiries can be directed to the corresponding author.
